# Adjusting ventilator settings to avoid air trapping in extremely premature infants reduces the need for tracheostomy and length of stay

**DOI:** 10.3389/fped.2022.1059081

**Published:** 2022-12-30

**Authors:** Ibrahim Sammour, Steven M. Conlon, Sarah E. Bauer, Gregory S. Montgomery, A. Ioana Cristea, Rebecca S. Rose

**Affiliations:** ^1^ Division of Neonatal-Perinatal Medicine, Department of Pediatrics, Indiana University, Indianapolis, IN, United States; ^2^ Division of Pulmonology, Department of Pediatrics, Indiana University, Indianapolis, IN, United States

**Keywords:** bronchopulmonary dysplasia, BPD, ventilator, interdisciplinary, team, tracheostomy, length of stay, outcome

## Abstract

Despite the improving understanding of how lung mechanics and tidal volume requirements evolve during the evolution of bronchopulmonary dysplasia (BPD), clinical management continues to be heterogeneous and inconsistent at many institutions. Recent reports have examined the use of high tidal-volume low respiratory rate strategies in these patients once disease has been well established to help facilitate their eventual extubation and improve their long-term neurodevelopmental outcomes. In this retrospective observational research study, we describe how intentional adjustment of ventilator settings based on patient lung mechanics by an interdisciplinary BPD team improved the care of the at-risk population of infants, reduced the need for tracheostomies, as well as length of stay over a period of over 3 years. The team aimed to establish consistency in the management of these children using a high tidal volume, low-rate approach, and titrating PEEP to address the autoPEEP and bronchomalacia that is frequently observed in this patient population.

## Introduction

In an effort to reduce the incidence and severity of BPD, lung protective strategies have been employed to stem the development of ventilator induced lung injury; a major contributor to the development of BPD (1–8). These early interventions, along with postnatal corticosteroid use and early extubation to CPAP have impacted the development of chronic lung disease in animal models and clinically in premature infants (9–13).

As lung architecture changes under the influence of prematurity, oxygen exposure, and inflammatory mediators that could be incited by sepsis, necrotizing enterocolitis, or ventilator induced lung injury, lung mechanics change significantly. While initial tidal volume targets of 4.5–6 ml/kg (14) are used by many Neonatal Intensive Care Units (NICU), it is known that anatomical dead space increases significantly with prolonged ventilation, this is likely contributed to by the development of tracheomegaly (15–18). This increase in dead space is often accompanied by rising lung compliance as the alveoli lose elastic recoil from alveolar simplification, and increasing airway resistance as the airways develop epithelial lesions and bronchomalacia. These changes lead to a rise in tidal volume requirements to maintain ventilation and avoid atelectasis, along with prolongation in the inspiratory and expiratory time constants of the lung. Continued use of a high ventilator rate in the setting of prolonged expiratory time constants leads to air trapping, causing hyperinflation of the lung and volutrauma despite smaller tidal volumes (4, 19, 20). If air trapping is not addressed by altering ventilator strategies, patients develop severe hyperinflation often leading to inability to wean from invasive mechanical ventilation.

The use of an open lung strategy, providing adequate tidal volume and addressing lung mechanics has been shown to reduce inflammatory mediators in animal studies, and resulted in lower pressure and oxygen requirements in clinical studies (6, 21–24). As lung disease is established and obstructive lung mechanics are more prominent, some patients develop hyper-inflation with increasing lung volumes which can lead to challenges for providing respiratory support (25).

Interdisciplinary programs lead by physicians who understand the changing mechanics and the complex care these infants require may improve outcomes. A hallmark of ventilator management in this group is the use of higher-tidal volumes, lower ventilator rates, and enough PEEP to address triggering and bronchomalacia that children with severe bronchopulmonary dysplasia experience (26–28). Our institution's rate of tracheosomy in preterm infants was higher than the 5% found by Padula et al. from data from the Childrens's Hospital Neonatal Consortium (29), but within the wide range of rates reported by Guaman et.al, from the BPD collaborative. Infants discharged from our hospital with tracheostomy and prolonged mechanical ventilation have an average of 2 hospital admissions and 16% mortality in the first year after initial discharge (30). Thus we aimed to decrease the rate of tracheostomy and prolonged mechanical ventilation in our patients at high risk of severe BPD or death.

This report describes the effect of an interdisciplinary BPD team utilizing individualized ventilator management directed at ventilating all of the lung while avoiding air trapping on the proportion of inborn premature infants at high risk for severe BPD or death who required tracheostomy.

## Methods

Our NICU is a level IV referral NICU offering subspecialty surgical care, and Extra Corporeal Life Support. Approximately one third of our patients are inborn at our level IV maternity center with most of the remaining patients coming from other level III NICUs around the state. We attempt to limit invasive mechanical ventilation in our preterm infants as much as possible while still providing adequate oxygenation and ventilation and supporting optimum growth. Despite these efforts, many patients remain on invasive mechanical ventilation at 28 days of age.

We use the Hamilton-G5^©^ (Hamilton Medical, Switzerland) in the neonatal mode for conventional ventilation in our NICU. The standard in our NICU is to use uncuffed endotracheal tubes (ETT) for the majority of patients on invasive mechanical ventilation, using the weight ranges recommended by NRP. However, chronically ventilated patients develop leaks around their ETTs with growth and stretching of the airway from the positive pressure of the ventilator. When a leak around the ETT is greater than 35% and interfering with patient/ventilator synchronicity, we consider upsizing the ETT if the patient's estimated dry weight is within 100 grams of the weight for the next size ETT. If the patient's estimated dry weight is within 500 grams but not 100 grams of the next larger ETT, we discuss the use of a cuffed ETT with the neonatal team. We only use cuffed tubes that have a Murphy eye, due to concern for acute obstruction when a Murphy eye is not present, thus the smallest cuffed tube we use is a 3.0 ID ETT. Patients with <35% leak around the ETT are generally placed in the volume targeted pressure control mode. Patients with >35% leak who are too small for a larger size or cuffed ETT are placed in conventional pressure control mode. We prefer assist control (CMV on the G5) for most patients to maximize ventilator synchrony and support growth by fully supporting every breath. Some patient are switched to SIMV plus pressure support during weaning or physician preference.

In our NICU, patients at high risk for BPD or death may have any of twenty-six neonatologists as the leader of their primary care team. At the initiation of this project, the primary neonatologist changed every 3 weeks, and in the middle of the project, staffing models changed and the primary neonatologist now changes every 2 weeks. Patients with long lengths of stay may have up to six different primary neonatologists. Frequently, ventilator strategies changed with each change of primary neonatologist. Before the development of the BPD team the pulmonologists were only consulted if the primary team felt the patient required investigation of airway disease with dynamic bronchoscopy, needed testing for pulmonary interstitial disease, needed a tracheostomy, or was diagnosed with pulmonary hypertension. Most patients were continued on the lower tidal volume higher rate strategy on the ventilator until extubated or transferred to the Pediatric Intensive Care (PICU) for transition to home ventilator after tracheostomy. Decision to place tracheostomy was made by the current attending neonatologist but was generally discussed with the parents when a patient over 40 weeks PMA was unable to be weaned on the ventilator and successfully transitioned to non-invasive respiratory support.

Our interdisciplinary BPD team was established to reduce variation in care both between patients and within the same patient throughout a long length of stay, improve transitions to our PICU and to the outpatient BPD clinic, decrease the number of patients requiring tracheostomy, and decrease hospital length of stay (LOS). An integral part of achieving these goals is the early collaboration between neonatologists and pulmonologists committed to improving the care of patients with BPD. This manuscript describes one focus of the BPD team to ameliorate progression of disease in the patients at highest risk for death or severe BPD. The inclusion criteria for a BPD team consult are that patients are born at <32 weeks estimated gestational age (EGA), and still require invasive mechanical ventilation at 28 days of life. Patients are seen by the BPD team as soon as they meet the inclusion criteria, which can be as early as 27 weeks post menstrual age (PMA). The team meets weekly along with the primary neonatologist caring for the patients. Patients are seen at least every other week, and weekly when not improving with interventions. Team members are available for questions from the primary team on request. Decisions about tracheostomy are discussed during BPD rounds, but the indications for pursuing tracheostomy have not changed.

During BPD rounds, clinical course, current chest imaging, trends in ventilator settings, FiO_2_, and respiratory rate are reviewed. Neonatologists and pulmonologists combine their expertise to develop and discuss recommendations for ventilator settings with the primary team. The goal of these recommendations is to avoid air trapping by ensuring adequate PEEP, avoid excessive respiratory rates, and ensure complete exhalation. Patients with signs of air trapping on chest x-ray (CXR) or requiring >70% FiO_2_ despite moderate to high ventilator settings get a patient-ventilator bedside assessment. Goals of this assessment are to ensure optimized PEEP setting and ensure adequate minute ventilation without relative tachypnea.

Our neonatologists on the BPD team, with input from the pulmonologists, developed a process for ventilator assessment (Table 1). At the bedside, with the patient in a calm state, dynamic pressure-volume loops and flow-volume loops are evaluated for increased inspiratory and expiratory flow resistance. Flow scalars are evaluated to ensure that full exhalation occurs with a brief period of expiratory pause before the next inhalation breath. If the resting respiratory rate exceeds 30 bpm, tidal volume target or set pressure control is gradually increased until a resting respiratory rate <30 bpm is achieved. Careful observation to ensure that all patient trigger efforts are detected by the ventilator is assessed by examining patient/ventilator interactions. If missed triggers are noted, and trigger sensitivity is appropriately set, it is likely that autoPEEP is interfering with the sensor's ability to detect the patient's attempts to breathe. To minimize autoPEEP, the PEEP is increased until patient ventilator synchronicity is reestablished (31, 32). Pressure-Volume loops and Flow-Volume loops are then assessed for improvement in obstructive patterns. Frequently, improvements in oxygen saturation will occur during these changes. If there are still concerns for autoPEEP, an expiratory hold maneuver is performed without routine administration of neuromuscular blockade. If it suggests autoPEEP is occurring, PEEP will be increased until improved, or until PEEP is increased to 3 cm H_2_O above the previous setting. In the event there is still concern for autoPEEP and large airway malacia is suspected, dynamic bronchoscopy through the ETT for PEEP titration by our Pulmonology members is recommended. Flow scalars are examined for adequacy of I-time. If an inspiratory pause is not observed, there is no significant leak, and end-inspiratory flow is still significantly positive with CXR findings of heterogenous disease, the I-time is increased by 0.05–0.1 s to allow for either a larger tidal volume delivery in setpoint pressure control, or for lower driving pressures in adaptive volume targeted pressure control schemes. We generally limit the increase in I-time to 0.1 s but could increase farther as long as inspiratory flow does not reach zero, and the respiratory rate is low enough that the increased I-time does not compromise expiratory time and full exhalation is achieved before the next breath. Of note, when an adaptive volume targeted mode is used, the pressure limit is liberalized to allow for adequate tidal volume delivery.

**Table 1:** Process for ventilator assessment.

Process for Bedside Ventilator Assessment	
Assessment	Action
Ensure patient is in a calm state	If unable to achieve calm state, discuss best time to return for assessment with the bedside nurse and neonatology providers
Evaluate dynamic pressure-volume loops and flow-volume loops for increased inspiratory and expiratory flow resistance.	Take picture in HIPPA compliant mobile phone application and upload into patient chart as pre assessment loops
Observe resting respiratory rate, if >30 bpm, gradually increase tidal volume until <30 bpm	If resting respiratory rate is >30 bpm, gradually increase tidal volume until <30 bpm over 5–15 min. Do not increase by more than 25%.^a^
Evaluate flow scalers to ensure a brief expiratory pause before next attempted breath.	If no expiratory pause, gradually increase tidal volume until this is achieved
Check patient trigger sensitivity	If too high, set to appropriate level (generally 0.1–0.3 lpm in flow trigger mode)
Evaluate for patient trigger efforts not resulting in a ventilator supported breath (when triggering sensitivity is set appropriately)	This is frequently caused by autoPEEP, address this by gradually increasing PEEP until all patient efforts result in a patient breath.^b^
Re-evaluate Pressure-Volume loops, Flow-Volume loops, and flow scaler for improvement in obstructive patterns	If still concerning for autoPEEP, perform expiratory hold maneuver without neuromuscular blockade. If concerning for autoPEEP increase PEEP until improved.^b^
Examine Flow scalars for adequacy of I-time	If there is no inspiratory pause and end-inspiratory flow is still significantly positive with CXR findings of heterogenous disease, increase the I-time by 0.05–0.1 s
Assessment Complete	Take picture in HIPPA compliant mobile phone application and upload into patient chart as post assessment loops

^a^ Most patients are in assist control with the rate set lower than their resting respiratory rate to allow for best synchronization of the ventilator with patient efforts.

^b^ If PEEP is increased by 3 more than before the assessment and there is still concern for autoPEEP, maintain there and recommend dynamic bronchoscopy through the ETT for PEEP titration.

For this retrospective observational research study patients at high risk for severe BPD were defined as those born before 29 completed weeks of gestation, and still requiring invasive mechanical ventilation at 28 days of life for respiratory issues (FiO_2_ >30% or parenchymal disease visible on CXR). To assess the effect of early interventions to avoid air trapping, patients were excluded from this analysis if they were admitted to our NICU after 32 weeks PMA. IRB approval for data collection to guide improvement efforts was obtained. The BPD team was implemented in September 2018. Data were collected from 1/1/2017 to 12/31/21. Patients were separated into quarters by birthdate for adequate sample sizes in each. The first 7 quarters were considered pre-intervention, “before BPD team.”.

## Statistics

Control charts were created in Excel^©^ using the QI Macros^©^ add in. We compared the proportion of patients with BPD who went on to require a tracheostomy, measured the LOS, measured the PMA at discharge, and assessed the proportion of patients discharged home in room air before and after the BPD team was established. A two-sided *t* test using QI Macros^©^ was used. A *p*-value of <0.05 was considered statistically significant.

## Results

**Figure 1:**
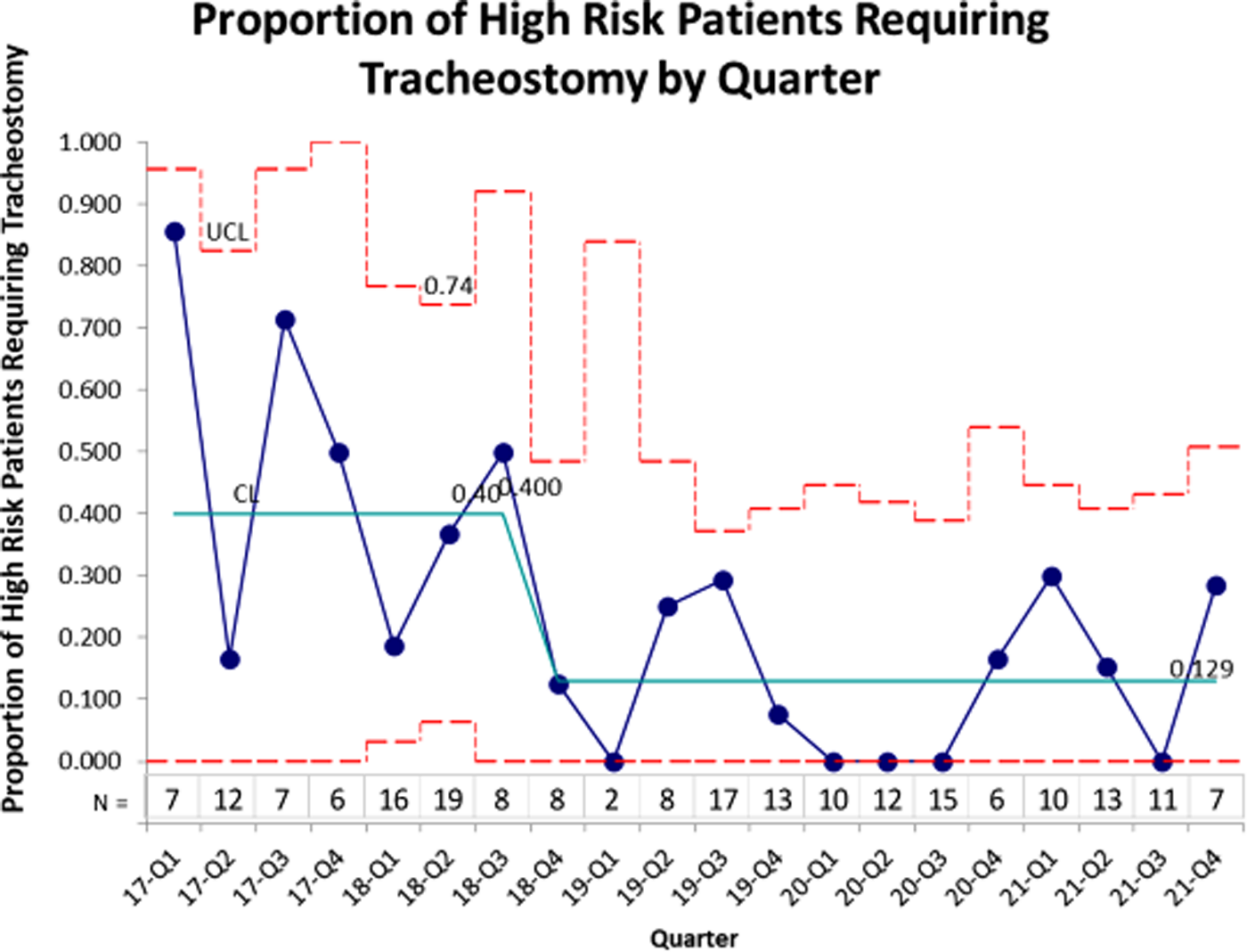
Proportion of patients at high risk for severe BPD requiring tracheostomy over time.

During the periods evaluated, 75 subjects met the criteria before the establishment of the BPD team, and 132 afterwards, for a total of 207 subjects. The EGA, age at admission, and PMA age at admission were similar in both periods (Table 2). The control chart for proportion of high-risk patients requiring tracheostomy is shown in Figure 1. The centerline from the 7 quarters prior to the BPD team was established was 40%. There was a rapid decline in the proportion of patients requiring tracheostomy after the BPD team, with all quarters having less than 40%. The centerline after this change shifted to 13%.

**Table 2:** Characteristics of patients at high risk for severe BPD.

	Before BPD Team	After BPD Team
Number of Patients	75	132
EGA at Birth, *mean (range)*	24.8 (22–28)	24.8 (22–28)
Age on Admission in Days, *mean (range)*	10 (0–62)	13 (0–61)
PMA at Admission, *mean (range)*	26.7 (22–31)	27.1 (22–31)

**Figure 2:**
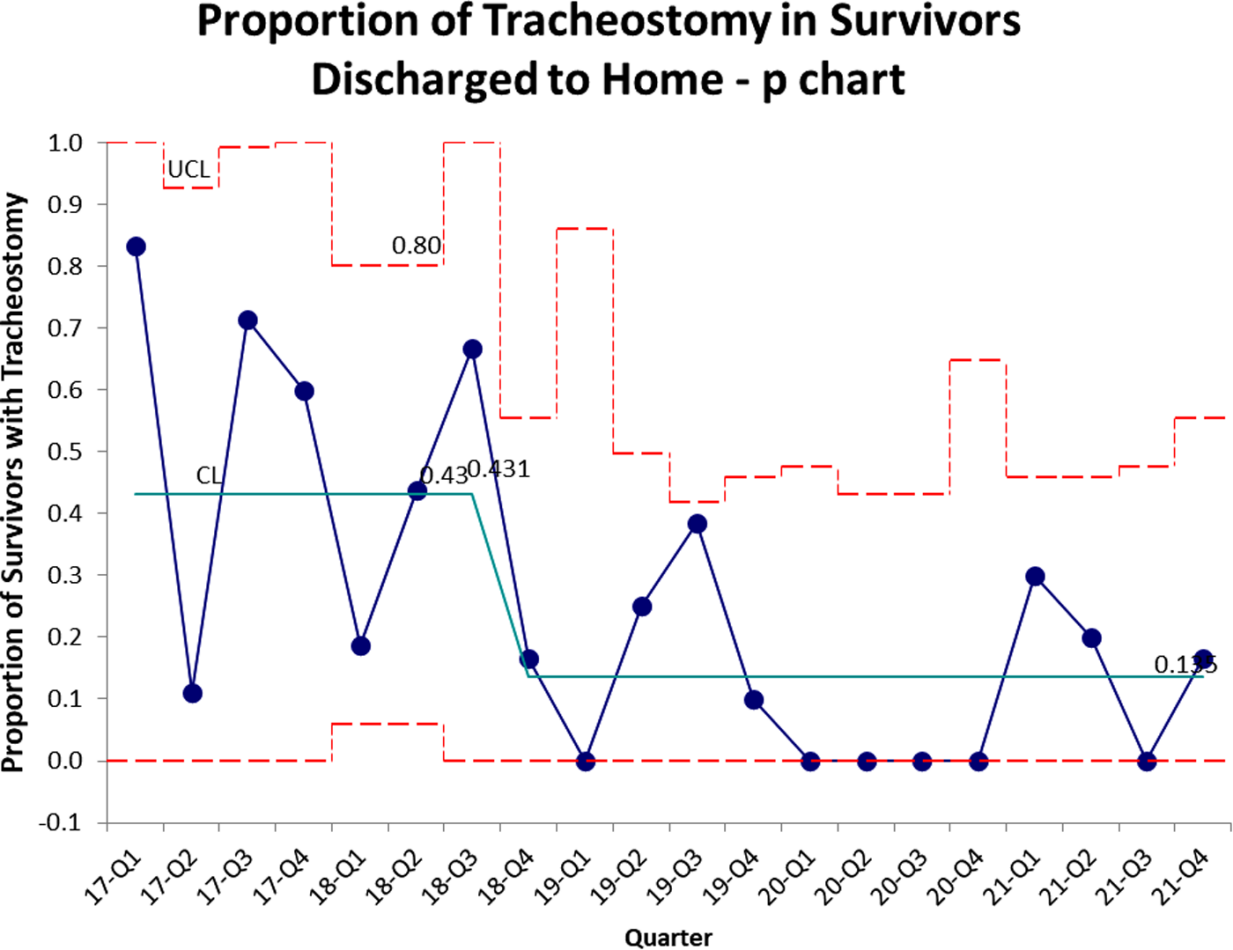
Proportion of survivors requiring tracheostomy.

The control chart of proportion of survivors requiring tracheostomy is shown in Figure 2. There is a similar decrease compared to Figure 1, with the mean decreasing from 43% to 12.5%.

**Figure 3:**
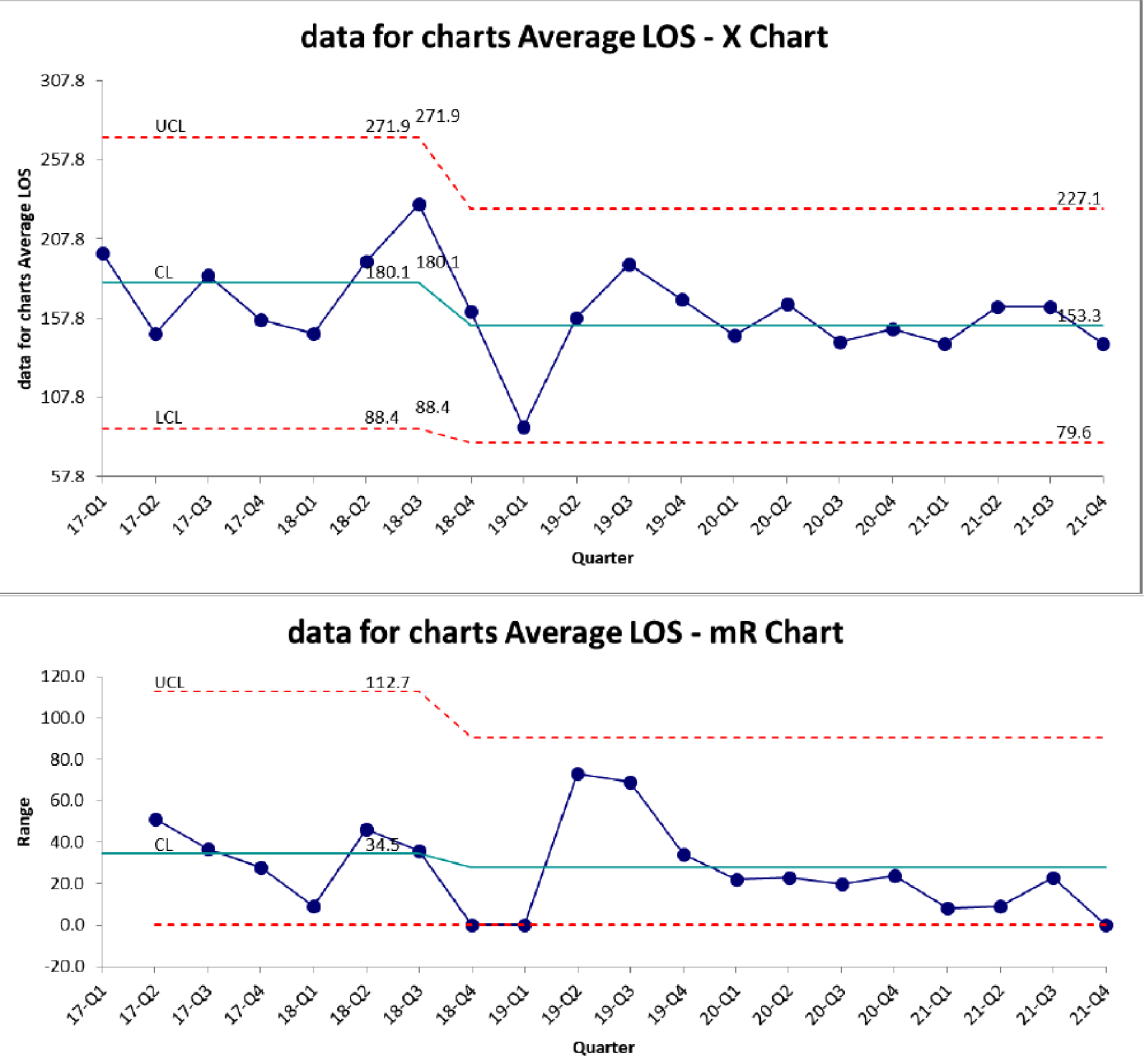
Average length of stay each quarter XmR chart.

Figure 3 demonstrates the control chart for length of stay. The mean LOS in the baseline period was 180 days. This decreased to 153 after the BPD team intervention but did not meet statistical significance. There is a trend towards decreased variation in LOS (Figure 3). PMA at discharge was not significantly different between the two epochs (Table 3). There was a trend towards more patients discharged home in room air, but it was not statistically significant (Table 3). We also evaluated the PMA at time of tracheostomy procedure (Table 3) as a measure of possible changes in decision making leading to tracheostomy between the two epochs. The mean PMA at the time of tracheostomy placement was not significantly different between the two epochs.

**Table 3:** Characteristics of patients at discharge.

	Before BPD Team	After BPD Team	*p*-value
PMA at Discharge (Weeks)	51.85	50.53	0.365
Percent Discharged in Room Air	5	13	0.089
Mean PMA at Time of Tracheostomy Procedure (Weeks)	47.49	48.87	0.360

## Discussion

Despite advances in the utilization of non-invasive support modalities to reduce the need for invasive support, a significant proportion of premature neonates continue to require prolonged invasive mechanical ventilation. This is associated with ventilator induced lung injury, especially atelectotrauma and volutrauma, when the changes in tidal volume and PEEP requirements are not addressed in this vulnerable population. Previous work had shown that an interdisciplinary team can improve long-term outcomes for these patients, however, did not address the timing of such interventions to reduce the need for tracheostomy (26, 27, 33).

In this this retrospective observational research study, we describe how early intervention by an interdisciplinary team focused on an open-lung strategy, addressing lung mechanics and patient-ventilator interactions can reduce the need for tracheostomy, improve LOS, and trend towards more patients being discharged home in room air.

Our interdisciplinary BPD team consults on patients earlier than previously described in the literature with the goal of early intervention in the patients most at risk for severe BPD and death. This earlier intervention may decrease variation in ventilation strategies by having the same team involved in the care throughout the NICU stay. In addition, our approach of adjusting ventilator settings based on the patients current lung physiology may ameliorate the progression of disease despite long term invasive ventilation. Following the initiation of the team-based intervention, we observed a significant decline in tracheostomy rates which did not come at the cost of a longer LOS or mortality. On the contrary, there was an improvement in the length of stay since the initiation of the program.

Our findings highlight the importance of addressing the changing lung mechanics and maintaining an open-lung approach in breaking the cycle of ventilator induced lung injury associated with more severe forms of bronchopulmonary dysplasia; specifically targeting atelectotrauma and abnormal patient-ventilator interactions. This requires accurate feedback from the conventional mechanical ventilator to facilitate the interpretation of the scalars, pressure-volume, and flow-volume loops. Our interdisciplinary group utilizes a bedside ventilator assessment to guide changes in ventilator parameters to minimize autoPEEP while addressing restriction to inspiratory and expiratory flow with larger tidal volumes and slower respiratory rates. These strategies have been recommended by multiple experts in the field (34, 35).

The goals of the ventilator assessment are based on the concept of addressing pathophysiologic changes in the lung utilizing information from the ventilator (32, 36). The restriction of flow during expiration often seen in BPD requires increased time for expiration before the start of the next breath. Thus the initial part of the assessment involves ensuring that the respiratory rate is slow enough to provide time for a complete expiration (26, 34). If the flow scaler depicts a brief pause in expiration before the next breath, there is less risk of autoPEEP (32, 36). The lack of a pause in expiration can be addresses by small incremental increases in tidal volume to decrease the patient initiated respiratory rate until a pause is seen.

Patient/ventilator asynchrony, demonstrated by patient efforts that do not result in a ventilator delivered breath despite appropriate trigger sensitivity, can be a sign of autoPEEP (31). When present, incremental increases in the PEEP to a level higher than the autoPEEP will improve patient/ventilator synchronicity and often decreases patient agitation. For patient safety, our practice is to only increase PEEP by 3 cm before stopping and reassessing over time, with consideration of dynamic bronchoscopy through the ETT for further PEEP titration.

Limitations of this study include a relatively small number of patients and it only represents care at one institution, with one brand of ventilator. In our NICU, as in many academic NICUs, there are several quality improvement projects and clinical research projects occurring concurrently. These concurrent projects may have influenced our results, although none of the projects were addressing this specific population. In addition, this project only addresses the short term outcomes of tracheostomy and LOS. As more patients are evaluated and managed by this interdisciplinary team on the inpatient and outpatient side, we are hoping to garner a better understanding of whether such early interventions also lead to improved long-term outcomes from a respiratory and neurodevelopmental standpoint. Finally, the consistent care plan from the interdisciplinary BPD team throughout the NICU stay may have contributed to the improvement in short term outcomes.

In summary, in our NICU, early intervention in premature infants at high risk for death or severe BPD, addressing lung mechanics and patient-ventilator interactions, with input from a dedicated BPD team, is associated with a decreased need for tracheostomy and decreased LOS. Our data support the need of a multi-center prospective study to further examine the effect of this type of ventilation approach in infants with high risk of death or severe BPD.

## Data Availability

The raw data supporting the conclusions of this article will be made available by the authors, without undue reservation.
